# Caveolin‐1 influences mitochondrial plasticity and function in hepatic stellate cell activation

**DOI:** 10.1002/cbin.11876

**Published:** 2022-08-16

**Authors:** Mariana Ilha, Leo A. Meira Martins, Ketlen da Silveira Moraes, Camila K. Dias, Marcos P. Thomé, Fernanda Petry, Francieli Rohden, Radovan Borojevic, Vera M. T. Trindade, Fábio Klamt, Florência Barbé‐Tuana, Guido Lenz, Fátima C. R. Guma

**Affiliations:** ^1^ Programa de Pós‐Graduação em Ciências Biológicas‐Bioquímica, Instituto de Ciências Básicas da Saúde Universidade Federal do Rio Grande do Sul – UFRGS Porto Alegre Rio Grande do Sul Brasil; ^2^ Department of Clinical Nutrition, Institute of Public Health and Clinical Nutrition University of Eastern Finland Kuopio Finland; ^3^ Departamento de Fisiologia, Instituto de Ciências Básicas da Saúde Universidade Federal do Rio Grande do Sul ‐ UFRGS Porto Alegre Rio Grande do Sul Brasil; ^4^ Departamento de Bioquímica, Instituto de Ciências Básicas da Saúde Universidade Federal do Rio Grande do Sul ‐ UFRGS Porto Alegre Rio Grande do Sul Brasil; ^5^ Departamento de Biofísica e Centro de Biotecnologia Universidade Federal do Rio Grande do Sul ‐ UFRGS Porto Alegre Rio Grande do Sul Brasil; ^6^ Centro de Medicina Regenerativa Faculdade Arthur Sa Earp Neto ‐ Faculdade de Medicina de Petrópolis Rio de Janeiro Brasil; ^7^ Programa de Pós‐Graduação em Biologia Celular e Molecular Escola de Ciências da Pontifícia Universidade Católica do Rio Grande do Sul‐ PUCRS Porto Alegre Rio Grande do Sul Brasil; ^8^ Centro de Microscopia e Microanálise Universidade Federal do Rio Grande do Sul ‐ UFRGS Porto Alegre Rio Grande do Sul Brasil

**Keywords:** caveolin‐1, hepatic stellate cell, liver fibrosis, lysosomal activity, mitochondrial cholesterol, mitochondrial plasticity

## Abstract

Caveolin‐1 (Cav‐1) is an integral membrane protein present in all organelles, responsible for regulating and integrating multiple signals as a platform. Mitochondria are extremely adaptable to external cues in chronic liver diseases, and expression of Cav‐1 may affect mitochondrial flexibility in hepatic stellate cells (HSCs) activation. We previously demonstrated that exogenous expression of Cav‐1 was sufficient to increase some classical markers of activation in HSCs. Here, we aimed to evaluate the influence of exogenous expression and knockdown of Cav‐1 on regulating the mitochondrial plasticity, metabolism, endoplasmic reticulum (ER)‐mitochondria distance, and lysosomal activity in HSCs. To characterize the mitochondrial, lysosomal morphology, and ER‐mitochondria distance, we perform transmission electron microscope analysis. We accessed mitochondria and lysosomal networks and functions through a confocal microscope and flow cytometry. The expression of mitochondrial machinery fusion/fission genes was examined by real‐time polymerase chain reaction. Total and mitochondrial cholesterol content was measured using Amplex Red. To define energy metabolism, we used the Oroboros system in the cells. We report that GRX cells with exogenous expression or knockdown of Cav‐1 changed mitochondrial morphometric parameters, OXPHOS metabolism, ER‐mitochondria distance, lysosomal activity, and may change the activation state of HSC. This study highlights that Cav‐1 may modulate mitochondrial function and structural reorganization in HSC activation, being a potential candidate marker for chronic liver diseases and a molecular target for therapeutic intervention.

## 1 INTRODUCTION

Chronic liver diseases account for 2 million deaths per year worldwide (Asrani et al., 2019). Regardless of the etiology of liver injury, the increased extracellular matrix (ECM) deposition by activated hepatic stellate cells (HSCs) is a common pathophysiological mechanism designating cells to fibrosis. Conceptually, the activation of HSCs is characterized by the transdifferentiation of quiescent, vitamin‐A‐storing cells into proliferative fibrogenic myofibroblasts (Singh et al., 2016). A variety of mechanisms mediate the HSCs pathway to activated states including a high requirement of energy, oxidative stress, endoplasmic reticulum (ER) stress, inflammation, apoptosis, and expression of proteins such as type I collagen and α‐smooth muscle actin (α‐SMA) (Trivedi et al., 2021; Tsuchida & Friedman, 2017). These mechanisms are tightly influenced by mitochondrial dysfunction due to a loss of mitochondrial flexibility (Longo et al., 2021).

Caveolin‐1 (Cav‐1) is an integral membrane protein of 22 kD (Parton & del Pozo, 2013) that exerts a critical role for interorganelle communication like ER and mitochondria (Fridolfsson et al., 2014). For instance, cholesterol is incorporated into the mitochondria membranes through specialized extensions of ER enriched in Cav‐1, named mitochondrial‐associated membranes (MAM) (Sala‐Vila et al., 2016). Also, cholesterol and Cav‐1 can regulate the mitochondrial dynamics (rate of fission and fusion) (Tilokani et al., 2018), and the lysosomal activity (Martin et al., 2016; Silva et al., 2020). Genetic ablation of Cav‐1 has been shown to increase the cholesterol content and to reduce mitochondrial respiration in hepatocytes and mouse embryonic fibroblasts (MEFs) (Fernandez‐Rojo & Ramm, 2016). It is known that reduced coupling between oxygen consumption and ATP synthesis leads to increased production of reactive oxygen species, apoptosis, and energy defects, which are signals that impact HSCs activation (Pesta & Gnaiger, 2012; Trivedi et al., 2021). In vitro studies with human and rat HSCs showed a mitochondrial bioenergetic signature that distinguishes activated HSCs from quiescent and less‐active HSCs (Gajendiran et al., 2018; Smith‐Cortinez et al., 2020). Moreover, Cav‐1 null MEFs displayed mitochondrial bioenergetic defects (Volonte et al., 2016) and premature senescence (Yu et al., 2017). Finally, Cav‐1 expression is increased in both sinusoidal endothelial cells and HSC of cirrhotic livers, showing the relevance of this protein in the development of chronic liver diseases (Yokomori et al., 2019, 2002).

In recent work, we developed a new HSC model by inducing exogenous expression of Cav‐1 in the GRX cell line, named GRX^EGFP‐Cav1^. This GRX cell line (Borojevic et al., 1985) is a relevant model to study the cellular mechanism of liver fibrosis once it can display an HSC quiescent‐like phenotype (Bitencourt et al., 2012; de Mesquita et al., 2013; Elias et al., 2019; Margis et al., 1992) or HSC activated‐like phenotype which is dependent on the environmental challenges (Guimaraes et al., 2006). We showed that exogenous expression of Cav‐1 was sufficient to induce classical parameters of activation, which highlights the potential role of Cav‐1 as a molecular effector of HSC transdifferentiation (Ilha et al., 2019). However, there are still gaps to fill regarding whether the expression of Cav‐1 interplay with parameters associated with the mitochondrial bioenergetic and dynamics and interorganelle communication, here namely as mitochondrial plasticity.

In light of these considerations, we hypothesized that Cav‐1 can regulate the mitochondrial bioenergetics and dynamics, and interorganelle communication in HSCs activation.

## 2 MATERIALS AND METHODS

### 2.1 Cell culture

The GRX cell line was established by Borojevic et al. (1985) and was kindly provided by the Cell Bank of Rio de Janeiro (HUCFF, UFRJ). Cells were routinely maintained in Dulbecco's modified Eagle's medium (Invitrogen) supplemented with 5% fetal bovine serum and 2 g/L HEPES buffer, gentamicin 50 µg/ml, fungizone 250 µg/ml, pH 7.4, at 37°C and 5% CO_2_.

### 2.2 Induction of Cav‐1 knockdown

The GRX^EGFP‐Cav1^ cell line which constitutively overexpresses 83% more Cav‐1 protein and GRX^EGFPpCineo^, which contain the enhanced green fluorescence protein (EGFP) control used in this study were previously established by our group (Ilha et al., 2019).

Short hairpin RNA (shRNA) lentiviral vectors pLKO.1‐NEO‐CMV‐TurboGFP™shCav1 were used to silence CAV‐1 with the lentiviral transduction particles from the Mission shRNA library from Sigma‐Aldrich (clone NM_007616.2‐487s1c1). Mission shRNA nontarget (Sigma Aldrich; pLKO.1‐neo‐CMV‐tGFP) was used as a nonsilencing control. GRX^GFPshCav1^ and GRX^GFPNT^ cells were generated by lentiviral infection and silenced cells were selected with G‐418 (Sigma‐Aldrich; G8168) (1000 μg/ml) to generate stable shRNA‐expressing pools, as previously described (Thome et al., 2016).

### 2.3 Ultrastructural analysis through transmission electron microscopy

#### 2.3.1 Cell preparation

Cells were fixed in 4% paraformaldehyde plus 2.5% glutaraldehyde in 0.1 M phosphate‐buffer solution (PBS). Afterward, the samples were fixed in 1% osmium tetroxide solution (Sigma Aldrich), gradually dehydrated in acetone (Merck), and soaked in epon resin. Ultrafine cuts were obtained and counterstained with 1% uranyl acetate (Merck) and with 1% lead citrate (Merck). Ultrastructural imaging was obtained by transmission electron microscopy (TEM; JEM 1200 EXII; Jeol) at an 80‐kV acceleration voltage (Ilha et al., 2019).

#### 2.3.2 Measurement of mitochondrial area (Â), shape *Z*, mitochondrial density, and ER‐mitochondria distance

Mitochondrial area (Â), shape *Z*, mitochondrial density, and ER‐mitochondria distance were measured in randomly selected fields of 15 images with 1 µm to 30k magnification as previously described (Lima et al., 2018). The mitochondrial area (*Â*) and perimeter (*P*) were measured by delineating mitochondria. The shape coefficient *Z* was used to quantify the mitochondrial elongation, using the following equation: Shape *Z* = *P*/√*Â*. Low shape *Z* values indicate more rounded mitochondria, while high values indicate more elongated mitochondria. The electron density of mitochondria was evaluated by measuring the grayscale of 8‐bits images (255 shades of gray). According to this method, 0 = is equivalent to absolute black and 255 = is equivalent to absolute white. The real distance between ER and outer mitochondrial membrane (OMM) was selected within 10–30 nm from ER and manually traced and quantified (images with 0.2 µm to 75k) by two blind researchers (Sala‐Vila et al., 2016). All the analyses were performed using the ImageJ software (NIH).

#### 2.3.3 Visualization of Cav‐1 through immunogold labeling

Samples were prepared as previously described (Wang et al., 2015). Then, samples were incubated with primary antibody against Cav‐1 (2:500, sc53564; Santa Cruz) and incubated with secondary antibody anti‐mouse conjugated to a gold particle of 9 nm (2:50, G7652‐4ml; Sigma‐Aldrich). Grids containing ultrathin sections were also analyzed after omitting the primary antibodies to test for the nonspecific reaction of the secondary colloidal‐gold conjugated antibody.

### 2.4 Laser‐scanning confocal microscopy analysis

#### 2.4.1 Evaluation of mitochondrial network and lysosomal content

The mitochondrial networks and lysosomal content were evaluated through staining live cells with Mitotracker® Red (Invitrogen Carlsbad) and Lysotracker Red DND 99 (LYRS) (Invitrogen) and were cultured in appropriate glass‐bottom culture plates (CELL view Glass bottom plates; Greiner Bio‐One). Images were collected using Olympus FV1000 laser‐scanning confocal microscopy at 37°C with humidified 5% CO_2_ air. Ten single confocal sections of 0.7 µM were taken parallel to the coverslip (*xy* sections) with an ×60 (numeric aperture 1.35) oil‐immersion objective (Olympus; U plan‐super apochromat, UPLSAPO60XO). For each sample, images of six fields were acquired and processed with Olympus FluoView FV1000 software. For LYSR, cell fluorescence intensity was analyzed using ImageJ (NIH; Ilha et al., 2019).

#### 2.4.2 Measurement of Cav‐1 protein through immunocytochemistry

For immunofluorescence labeling, cells were fixed with 4% paraformaldehyde before incubation with the primary antibody Cav‐1 (1:500; sc53564; Santa Cruz Biotechnology). Sequentially, cells were incubated with the secondary antibody Alexa Fluor 555 (1:1000; Invitrogen). All images were acquired in the Olympus FV1000 laser‐scanning confocal microscope as previously described. All experiments were performed at least four times for each sample. Images from six random fields were acquired, and cell fluorescence intensity was analyzed using ImageJ (NIH; Meira Martins et al., 2014).

### 2.5 Measurement of total and mitochondrial cholesterol from isolated mitochondria

To access the total and mitochondrial cholesterol from isolated mitochondria, cells were seeded in six‐well plates (15 × 10^4^ cells/cm²). After, cells were scratched in PBS and transferred to a precooled glass Potter homogenizer with lysis buffer (10 mM TRIS‐HCL, 0.25 M sucrose, 20 mM NaF, 1 mM dithiothreitol, 5 mM ethylenediaminetetraacetic acid, and protease inhibitor). Cells were homogenized with 7 min strokes at medium speed and centrifuged for 5 min at 100*g*, 4°C. Next, the supernatant was centrifuged at 4°C for 15 min at 10,000*g*. Then, the supernatant was stored (cytosolic fraction), while the mitochondrial pellet was resuspended in lysis buffer for cholesterol quantification (Amoedo et al., 2011). Mitochondrial and total cholesterol were measured using the Amplex Red Cholesterol Assay Kit (Invitrogen) following the manufacturer's instructions.

### 2.6 Measurement of gene expression through a quantitative real‐time polymerase chain reaction

Total RNA from 10^6^ cells was extracted using TRIzol reagent (Invitrogen) and was reverse‐transcribed with SuperScript‐II (Invitrogen). RNA expression levels were quantified by measuring the SYBR Green on Step One Plus real‐time cycler (Applied‐Biosystems). The geNorm software was used to indicate the most stable gene (Vandesompele et al., 2002). In this analysis, mitofusin 1 (MFN1) presents the most stable gene and was used as a constitutive gene. Samples were then analyzed using the $\Delta \Delta C_{t}$ method (Schmittgen & Livak, 2008). Gene sequence information was collected from free‐internet databases (www.ensembl.org and https://www.ncbi.nlm.nih.gov/refseq/) and used to design specific primers in (Table 1) using freely available software from Integrated DNA Technologies (www.idtdna.com) (Grun et al., 2018).

**Table 1:** Primers sequences for real‐time q‐PCR analyses of different genes

Gene	Primer sequence	GenBank reference	Amplicon
Cav‐1	F‐5′GCACACCAAGGAGATTGACC3′	NM 001243064	180 bp
	R‐5′GACAACAAGCGGTAAAACCAA3′		
DRP1	F‐5′TCAATAAGCTGCAGGACGTC3′	NM 001276340.1	197 bp
	R‐5′TTCTGGTGAAACCTGGACTAG3′		
RnR2	F‐5′AGCTATTAATGGTTCGTTTGT3′	MGI 102492	130 bp
	R‐5′AGGTGGCTCTATTTCTCTTGT3′		
MFN1	F‐5′GGTGGAAATACAGGGCTACAG3′	NM 024200.4	183 bp
	R‐5′ACACTCAGGAAGCAGTTGG3′		
OPA1	F‐5′ACGACAAAGGCATCCACC3′	NM 001199177.1	177 bp
	R‐5′GAGCAATCATTTCCAGCACAC3′		
αSMA	F‐5′CTTCGCTGGTGATGATGCTC3′	NM 007392.3	167 bp
	R‐5′TGATGCCGTGTTCTATCGGA3′		

Abbreviation: q‐PCR, quantitative polymerase chain reaction.

### 2.7 Flow cytometry

Mitochondrial function and lysosomal content were evaluated through staining cells with Mitotracker® Red (Invitrogen) and LYSR (Invitrogen), respectively. Briefly, cells were cultured in 12‐well plates, trypsinized, and incubated for 30 min with Mitotracker® Red or LYRS, following the manufacturer's instruction. All data (10,000 events) were acquired with a FACS cytometry system (FACS Calibur; BD Bioscience) controlled by Cell Quest software (BD Bioscience), then analyzed using FCS Express 4 Software (De Novo Software).

### 2.8 Measurement of cellular oxygen consumption through high‐resolution respirometry

Cellular oxygen consumption was measured by high‐resolution respirometry (HRR) in the Oroboros Oxygraph‐2k in standard configuration, with 2 ml final volume on both chambers, at 37°C, and 750 rpm stirrer speed. For all experiments, 0.8 million cells were used per chamber and the data was acquired in pmol of O_2_ per second per number of cells. The software DatLab 4 (Oroboros Instruments®, Innsbruck, Austria) was used for data acquisition. The protocol used consisted of the application of oligomycin (Sigma®), carbonyl cyanide‐4‐(trifluoromethoxy) phenylhydrazone (Sigma®), rotenone (Sigma®), and antimycin A (Sigma®) (Pesta & Gnaiger, 2012). The bioenergetic health index (BHI) was calculated accordingly (Chacko et al., 2014).

### 2.9 Statistical analysis

Samples were tested for normality using D'Agostino and Pearson tests. Data were obtained at least from three biological replicates and submitted to one‐way analysis of variance followed by Bonferroni's post hoc test. Statistical significance was accepted at *p* ≤ .05. Data are expressed as means ± standard deviations, as well as the number of experiments performed, and are indicated in each figure. All analyses were performed using the statistical software GraphPad Prism 6 for Windows (GraphPad Software Inc., version 6).

## 3 RESULTS

### 3.1 Cav‐1 expression was successful knocked down in GRX

Previously we established the GRX^EGFP‐Cav1^ cell line that constitutively expresses more than 83% Cav‐1 protein than GRX cells (Ilha et al., 2019). Here, we show that this increase in protein expression is related to the 150% more Cav‐1 messenger RNA (mRNA; Supporting Information: Figure S1A). Now, we stably knocked down the expression of Cav‐1. This new cell line led to a reduction of 64% Cav‐1 mRNA in GRX^GFPshCav1^ when compared with the endogenous level (GRX) (Supporting Information: Figure S1A). To confirm that the expression of green fluorescent proteins in GRX^GFPNT^ and GRX^EGFPpCineo^ was not altering the α‐SMA expression, a marker of HSC activation, we checked it by real‐time polymerase chain reaction (RT‐PCR; Supporting Information: Figure S1B). Indeed, no difference was found in the intensity of fluorescence of Cav‐1 protein content in GRX^GFPNT^ and GRX^EGFPpCineo^ when compared with GRX. However, GRX^GFPshCav1^ showed a significant decrease, corroborating also with the decrease of Cav‐1 mRNA expression (Supporting Information: Figure S1C,D). Altogether, the GRX^GFPshCav1^ model presented a successful knocked down of Cav‐1 expression.

### 3.2 Cav‐1 changes the mitochondrial morphology, ER shape, and ER‐OMM distance

**Figure 1:**
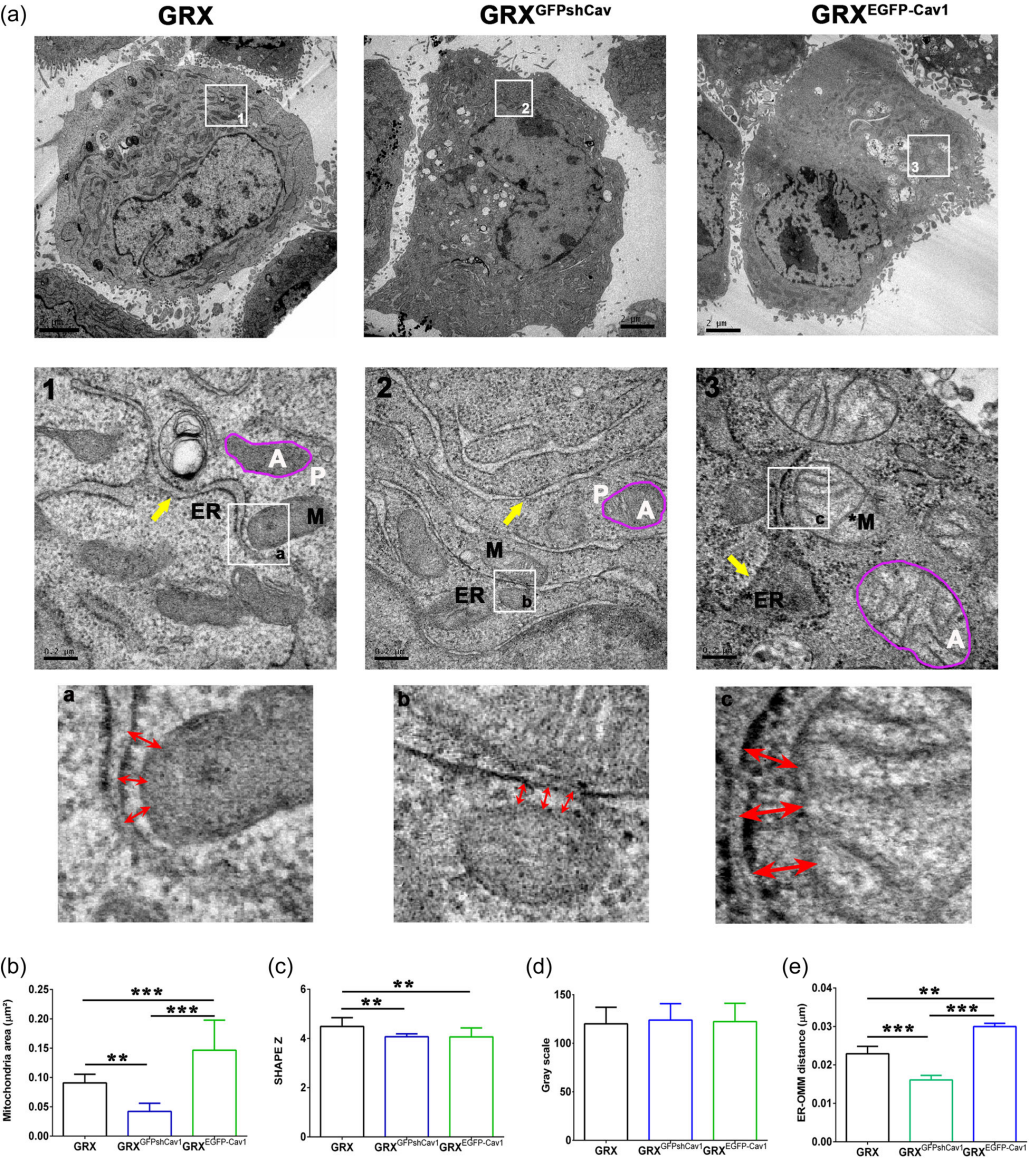
Caveolin‐1 (Cav‐1) changes the mitochondrial morphology, endoplasmic reticulum (ER) shape, and ER‐OMM distance (MAM). (a) Ultrastructural analysis revealed that both exogenous expression and knockdown of Cav‐1 changed organelle morphology: mitochondria (M), dilated mitochondria (M*), ER, dilated ER (ER*), white A: area, white P: perimeter. Red double‐edge arrows represent the distance between the outer mitochondrion membrane (OMM) and ER membrane (MAM). Yellow arrows show the dilated ER (GRX^EGFP‐Cav1^) or thin ER (GRX, GRX^GFPshCav1^). (b) The mitochondrial area in µm². (c) Shape *Z* coefficient (shape *Z* = *P*/√*Â*, where *P* is the perimeter and *Â* is the area). (d) Grayscale represents mitochondrial density. (e) ER‐OMM distance in µm by measuring the double‐edge red arrows. Data were measured by ImageJ and represented the mean ± SD from at least 15 images (*n* = 3 experiments, **p < .01; ***p < .001 such as indicated by one‐way ANOVA followed Bonferroni's post hoc test. ANOVA, analysis of variance; EGFP, enhanced green fluorescence protein; MAM, mitochondrial associated membrane; OMM, outer mitochondrial membrane.

To investigate the role of Cav‐1 expression on morphological changes in mitochondria, we focused on measuring key parameters of mitochondrial morphology at the ultrastructural level. GRX^EGFP‐Cav1^ cells presented mitochondria with a rounded shape, enlarged morphology, and clear but irregular crests (M*) (Figure 1a, insert 3). Another notable ultrastructural difference between GRX^EGFP‐Cav1^ and GRX^GFPshCav1^ cells was the ER morphology and its physical interaction/proximity with the OMM (Figure 1a, inserts a–c and Figure 1e) (Sala‐Vila et al., 2016). ER in GRX^EGFP‐Cav1^ appears to have a larger lumen (Figure 1a, insert 3, yellow arrows), and the physical ER‐OMM interaction is reduced (Figure 1a, insert c, red double edge arrows). On the other hand, ER in GRX^GFPshCav1^ is arranged in thin tubules (Figure 1a, insert 2, yellow arrows), and the physical ER‐OMM interaction seems to be higher (Figure 1a, insert b and Figure 1e, red double‐edge arrows).

Through morphometric analysis, we found that GRX^EGFP‐Cav1^ cells showed an increased mitochondrial area while GRX^GFPshCav1^ cells presented a decreased mitochondrial area (Figure 1b). The decrease in the Shape Z value in GRX^EGFP‐Cav1^ and GRX^GFPshCav1^ revealed that both cells presented more rounded mitochondria than GRX cells (Figure 1a, inserts 1–3, and Figure 1c). However, no difference was found in the electron density of mitochondria (Figure 1d), thereby indicating no changes in mitochondrial crest density. Through the Immunogold technique, we confirm the presence of Cav‐1 in the plasm membrane and observed labeled Cav‐1 in mitochondria, ER, MAM, and nuclear membrane of GRX and GRX^EGFP‐Cav1^ (see arrows in Supporting Information: Figure S2).

### 3.3 Cav‐1 alters the mitochondrial dynamics and cholesterol content

**Figure 2:**
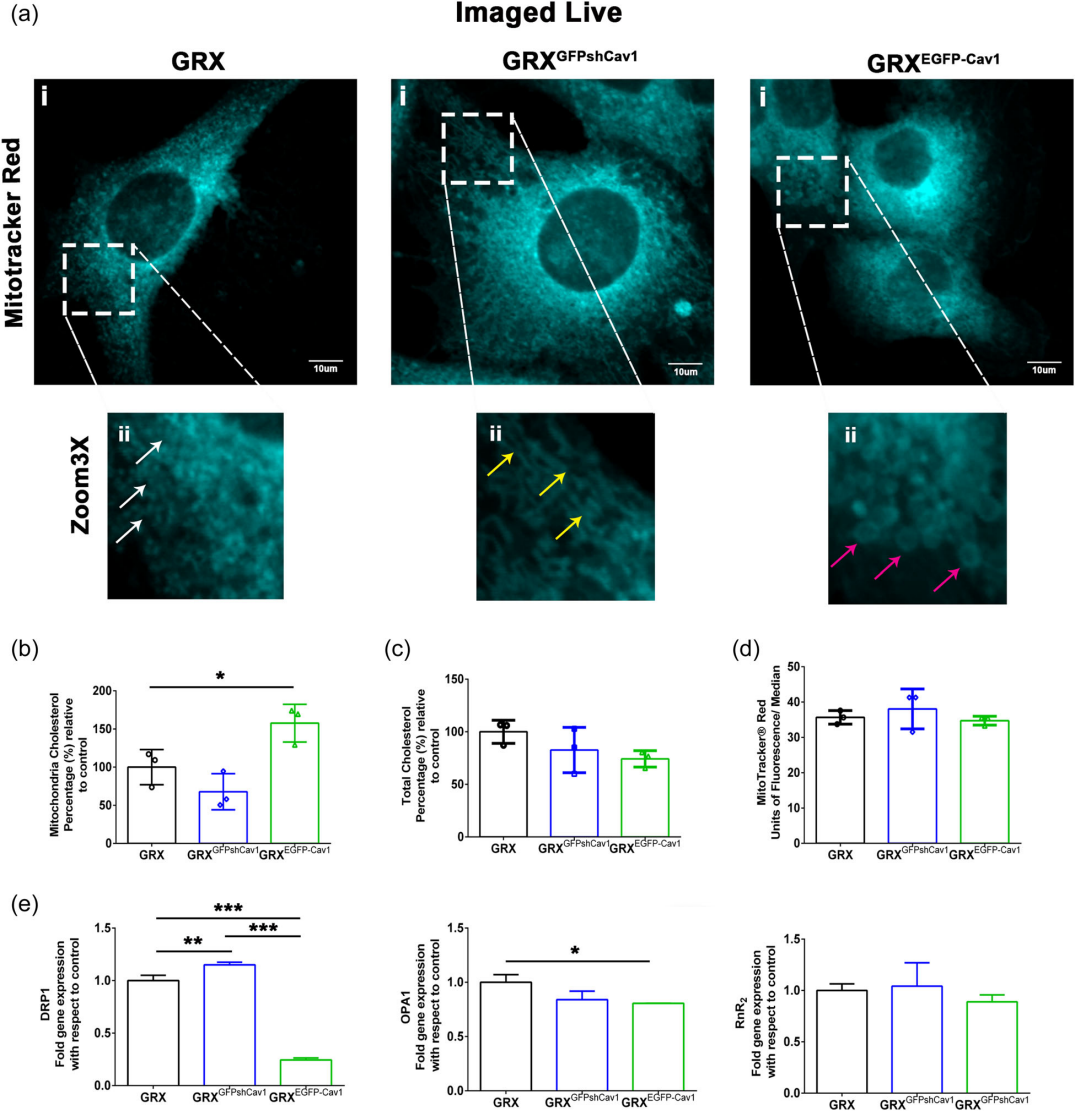
Caveolin‐1 (Cav‐1) alters mitochondrial dynamics and cholesterol content. (a) Representative confocal images of living GRX, GRX^GFPshCav1^, and GRX^EGFP‐Cav1^ cells showing the mitochondrial network stained by MitoTracker® Red. Images were pseudocolored in “cyan blue” using ImageJ. Arrows in ×3 zoom images indicate white, small, and round mitochondria; yellow, more elongated mitochondria; and pink, ring‐shaped mitochondria (*n*= 4, at least 15 images are acquired by the group. Scale bar = 10 µm). (b, c) Quantification of mitochondrial and total cholesterol was performed by Amplex Red. The values were shown in percentage (%) relative to control (GRX). (d) MitoTracker® Red analyses were performed by flow cytometry. (e) The mRNA expression of DRP1, OPA1, and RnR_2_. Graphs values are means ± SD (*n* = 3 experiments, *p < .05; **p < .01; and ***p < .001 such as indicated by one‐way ANOVA followed by Bonferroni's post hoc test). ANOVA, analysis of variance; DRP1, dynamin 1‐like protein; EGFP, enhanced green fluorescence protein; mRNA, messenger RNA; OPA1, optic atrophy protein 1; RnR_2_, RNA ribosomal 2.

Next, we labeled living cells with MitoTracker® Red for examining them by confocal microscopy and flow cytometry, intending to check if there were alterations in the mitochondrial dynamics and function (Figure 2a,d). GRX mainly presented a perinuclear mitochondrial network with punctual morphology (Figure 2a, insert ii, white arrows). However, GRX^EGFP‐Cav1^ cells mainly displayed larger and rounder mitochondria that also extend to the peripheral zone of the cell cytoplasm that forms a ring‐shaped mitochondria network (Figure 2a, insert ii, pink arrows). On the other hand, GRX^GFPshCav1^ cells presented thin mitochondrial tubules and individual dots that extend to the peripheral zone of the cell cytoplasm (Figure 2a, insert ii, yellow arrows). The high expression of Cav‐1 induced an increase in the mitochondrial cholesterol content (Figure 2b); nonetheless, it did not affect the cellular total cholesterol content (Figure 2c), nor the potentially mitochondrial function through measuring the fluorescence intensity of Mitotracker® Red by flow cytometry (Figure 2d).

Based on the final ranking of geNorm we found that MFN1 was the most stable gene (*M* = 0.563); thus, this gene was used as a constitutive normalizer in the RT‐PCR‐based experiments. Exogenous expression or knockdown of Cav‐1 led to important changes in genes responsible for the mitochondrial fusion and fission processes. GRX^EGFP‐Cav1^ cells presented a decrease in the dynamin 1 like (DRP1) and optic atrophy protein 1 (OPA1) mRNA expression while GRX^GFPshCav1^ cells showed an increase in the DRP1 mRNA expression. Furthermore, we found no changes in RNA ribosomal 2 (RnR_2_) expression, which is an important gene for mitochondria biogenesis (Figure 2e). Interestingly, through Pearson's analyses, it was revealed that mitochondrial cholesterol content correlated significantly with the mitochondrial area (*R* = 0.78; *p* = .0136) and perimeter (*R* = 0.77; *p* = .015), which may indicate that there is no change in the lipid composition of mitochondrial membranes. Also, we checked that mitochondrial cholesterol content correlates with ER‐OMM distance (*R* = 0.68; *p* = .045), Cav‐1, and DRP1 mRNA expression (*R* = 0.78; *p* = .013; *R* = −0.74; *p* = .022, respectively).

### 3.4 Cav‐1 modifies mitochondrial respiration

**Figure 3:**
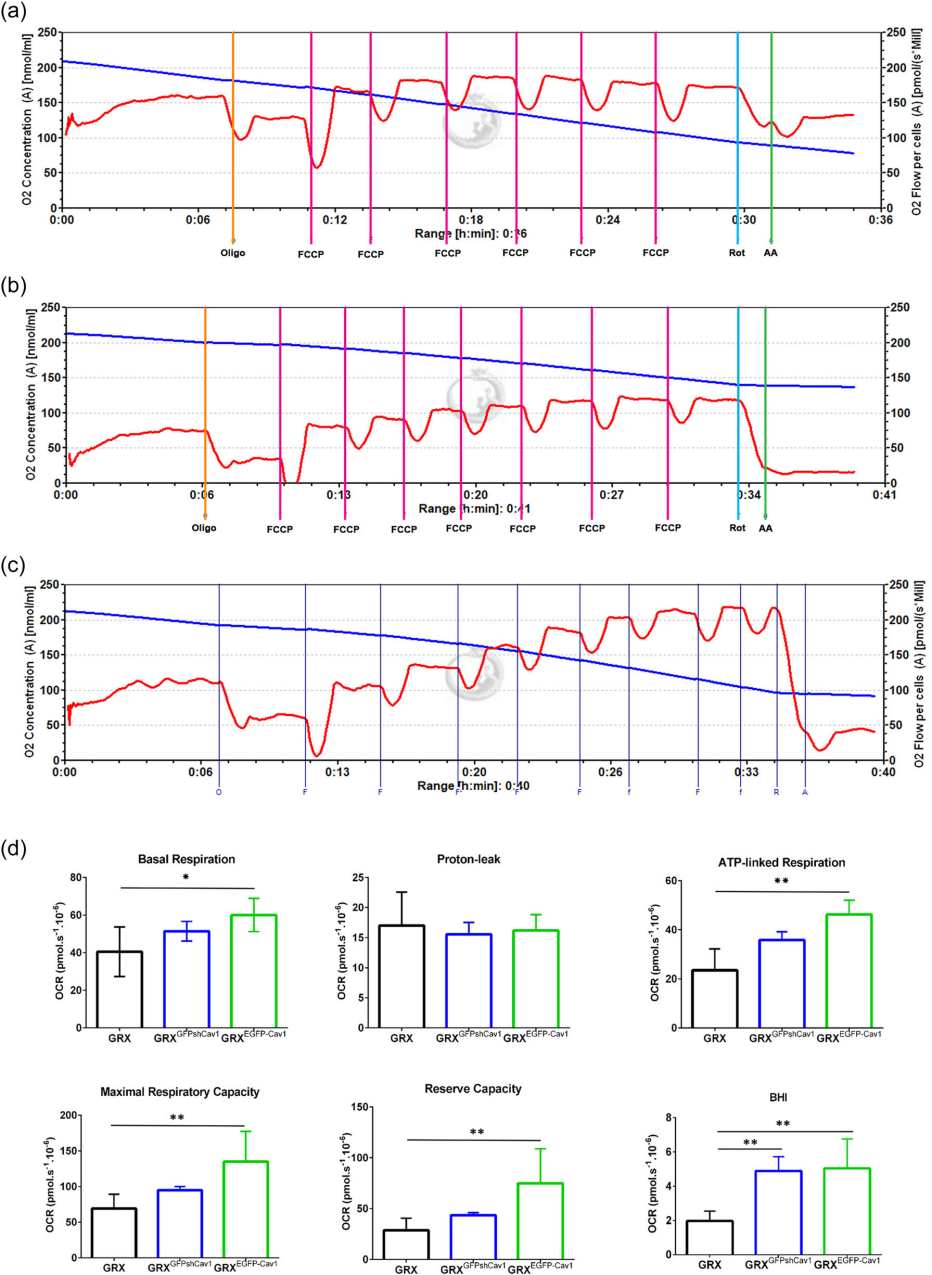
Caveolin‐1 modifies mitochondrial respiration. The representative curve for the high‐resolution respirometry performed in Oroboros Oxygraph‐2k for (a) GRX, (b) GRX^GFPshCav1^, and (c) GRX^EGFP‐Cav1^. The blue curve demonstrates the oxygen concentration in the sealed chamber and the red curve shows the oxygen consumption per million cells. The protocol includes the sequential application of Oligomycin (final concentration of 2 µg/ml, Oligo) followed by several times by 1 µl of 0.1 mM FCCP solution and finishing with the application of 0.5 µM rotenone (Rot) and 2.5 µM antimycin A (AA). (d) Exogenous expression of Cav‐1 increases basal and ATP‐linked respirations, maximum and reserve capacity, and bioenergetic health index (BHI). Knockdown of Cav‐1 increased the BHI. Proton leak was not affected in all groups. (at least *n* = 3 experiments, *p < .05 and **p < .01 such as indicated by one‐way ANOVA followed by Bonferroni's post hoc test). ANOVA, analysis of variance; FCCP, carbonyl cyanide‐4‐(trifluoromethoxy) phenylhydrazone; OCR, oxygen consumption rate.

To test whether Cav‐1 expression can directly influence components of mitochondria respiration and its physiology in HSC, we performed the HRR experiment in the OROBOROS Oxygraph‐2k equipment (Figure 3a–d). The blue curve demonstrates the oxygen concentration in the sealed chamber and the red curve shows the oxygen consumption per million cells of GRX, GRX^GFPshCav1^, and GRX^EGFP‐Cav1^ (Figure 3a–c). Our results demonstrated that exogenous expression of Cav‐1 increases Basal, ATP‐linked respirations, maximum and reserve capacity, and BHI. Curiously, the knockdown of Cav‐1 also increased the BHI in GRX^GFPshCav1^. Interestingly, the proton‐leak was not affected in all groups, thus indicating that the mitochondrial function was conserved regardless of the exogenous expression or knockdown of Cav‐1 (Figure 3d). This result confirms the analysis of mitochondrial function performed by Mitotracker® Red labeling (Figure 2d).

### 3.5 Exogenous expression and knockdown of Cav‐1 promoted features of HSC activation state

**Figure 4:**
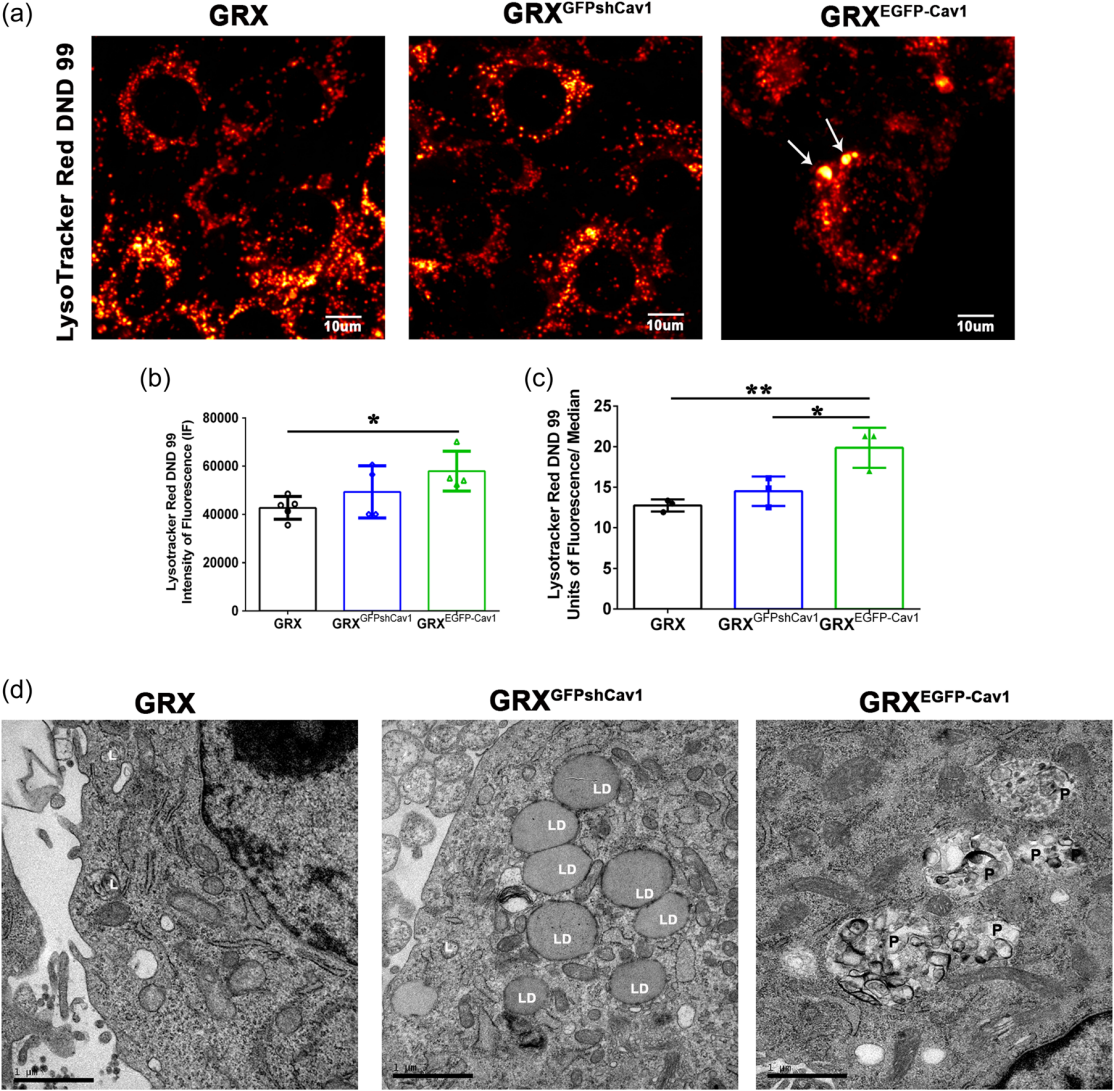
Exogenous expression and knockdown of Caveolin‐1 (Cav‐1) induce lysosomal activity and lipid droplets formation. (a) Representative images of lysosome after staining cells with LYSR of GRX, GRX^GFPshCav1^, and GRX^EGFP‐Cav1^ cells. White arrows showed enlarged lysosomes in GRX^EGFP‐Cav1^. The images were pseudocolored in “red hot” and measured (b) the intensity of fluorescence. Scale bar = 10 µm. (c) Lysosomal bioactivity was evaluated through staining cells with LYSR followed by flow cytometry (*n* = 3 experiments, *p < .05; **p < .01 such as indicated by one‐way ANOVA followed by Bonferroni's post hoc test). (d) Representative images of TEM of GRX, GRX^GFPshCav1^, and GRX^EGFP‐Cav1^ cells, when white L: lysosome, white LD: lipid droplet, black P: phagolysosome. *n* = 4 experiments at least 15 images are done which group. Scale bar = 1 µm. ANOVA, analysis of variance; EGFP, enhanced green fluorescence protein.

Cav‐1 coordinates the autophagic process to deliver cytoplasm compounds to lysosomes, a process that can interfere with mitochondrial dynamics and can drive HSC activation (Shi et al., 2015; Tsuchida & Friedman, 2017). Then, we analyzed the activity and network of the lysosomal compartment through flow cytometry and confocal microscopy after staining cells with LYSR. We showed that cells with exogenous expression of Cav‐1 presented LYSR stained vesicles with high fluorescence through confocal microscopy (Figure 4a,b, white arrows) and this increase in LYSR fluorescence was quantified by flow cytometry (Figure 4c). The presence of lysosome/phagolysosome (black P) was confirmed through TEM in GRX^EGFP‐Cav1^, a feature of a more activated stage of HSC (Figure 4d). Interestingly, the knockdown of Cav‐1 revealed a high number of lipid droplets (white LD), a feature of a less activated stage of HSC (Figure 4d). Moreover, Pearson's analyses also showed that mitochondrial cholesterol correlated significantly with LYSR (*R* = 0.72; *p* = .029).

## 4 DISCUSSION

Over the years, the efficiency of mitochondrial respiration, loss of mitochondrial plasticity, and increase of Cav‐1 expression in HSC have been considered critical for the progression of liver fibrosis (Longo et al., 2021; Yokomori et al., 2019, 2002). In this context, there is still interest in understanding the overall metabolic characteristics and energetics; when is the critical point that the mitochondria lose the flexibility and ability to support the damage, and how Cav‐1 has a role in the mitochondrial physiology of the HSC. During HSC activation, the increase of ECM synthesis by ER enlargement demands a high supply of intracellular energy. These cells undergo important metabolic changes that may interfere with their mitochondrial metabolism, dynamics, and organization (Gajendiran et al., 2018). In recent work, our research group demonstrated that exogenous expression of Cav‐1 was sufficient to induce GRX cells, to a more activated‐like state, by enhancing some classical markers of activation (Ilha et al., 2019). Here, we established the knockdown of Cav‐1 in GRX cells for creating the GRX^GFPshCav1^ cell line, a robust tool to understand the role of Cav‐1 in HSC. Considering our previous results, we investigate the influence of Cav‐1 on mitochondrial bioenergetic, dynamics, and inter‐organelle communication through modulating the mitochondrial plasticity in HSC activation.

The first question of this study sought to determine the actual contribution of exogenous expression or knockdown of Cav‐1 on mitochondrial plasticity, dynamics, and respiration in HSCs activation. Knockdown of Cav‐1 showed a smaller mitochondrial area and perimeter, more individual dots with thin mitochondrial tubules distributed along with the periphery of the cells, increase in DRP1 expression, similarly to less activated HSCs. However, exogenous expression of Cav‐1 demonstrated an increase in area and perimeter, a highly fused ring‐shaped mitochondrial network extended towards the cell periphery, decrease in DRP1 and OPA1 expression, equivalently with more activated HSCs (Friedman et al., 1992; Smith‐Cortinez et al., 2020). Mitochondria morphology is connected to its function: the fragmentation occurs in response to nutrient excess and the mitochondrial fusion is associated with increased cell bioenergetic demands (Leonard et al., 2015; Smith‐Cortinez et al., 2020). ER tubules and DRP1 are recruited to mitochondria to form wrapping around and constriction ring that drives the organelle fragmentation (Lopez‐Crisosto et al., 2017; Martin et al., 2016). On the other hand, OPA1 is present in the inner mitochondrial membrane (IMM) that coordinates the fusion manner (Song et al., 2015). Thereby, the exogenous expression or knockdown of Cav‐1 could interfere in the fission/fusion machinery balance and ER‐OMM distance, associated with mitochondrial architecture, respiration, and plasticity of HSC. Interestingly, the induction of mitochondrial OXPHOS by exogenous expression of Cav‐1 was accompanied by extensive mitochondrial fusion, without changes in mitochondrial biogenesis through RnR_2_ expression. On the other hand, the knockdown of Cav‐1 increases the fission process, and decreases the ER‐mitochondrial distance, without changing the metabolic demand and mitochondrial biogenesis. These results suggest that an increase in mitochondrial fusion or decrease of mitochondrial fission by exogenous expression of Cav‐1 is a direct response to the increased energetic demand during HSC activation (Trivedi et al., 2021).

Interestingly, we observed that alterations in Cav‐1 expression did not affect mitochondrial crest density but increased respiration when submitted to metabolic challenges. This characteristic is a feature of mitochondrial plasticity to adapt to the increase of electrons flow and OXPHOS production, driving to activation of HSCs in the early stages of liver damage (Longo et al., 2021). Oximetry analysis revealed that exogenous expression of Cav‐1 led to an increase in mitochondrial function without causing oxidative damage to the IMM and the electron transport chain (ETC) complexes. Furthermore, Cav‐1 could be a pathway for plasma membrane repair by preserving the membrane ultrastructure and mitochondrial function (Schilling & Patel, 2016). Another possible explanation for the increase in the parameters of mitochondria respiration in GRX^EGFP‐Cav1^ is the protective mechanism of Cav‐1 signaling in the formation of supercomplexes with the ETC. Importantly, dynamics changes in mitochondrial architecture can promote supercomplexes assembly as a regulatory mechanism for the bioenergetic adaptation to metabolic demand in HSC activation (Baker et al., 2019). However, whether the Cav‐1 has some influence on the formation of supercomplexes in mitochondrial respiration in HSC activation is still unknown (Lobo‐Jarne & Ugalde, 2018; Milenkovic et al., 2017). Thus, metabolic and flexibility shifts in mitochondrial plasticity by Cav‐1 associated with HSC activation report novel and potent targets for the treatment of liver fibrosis.

**Figure 5:**
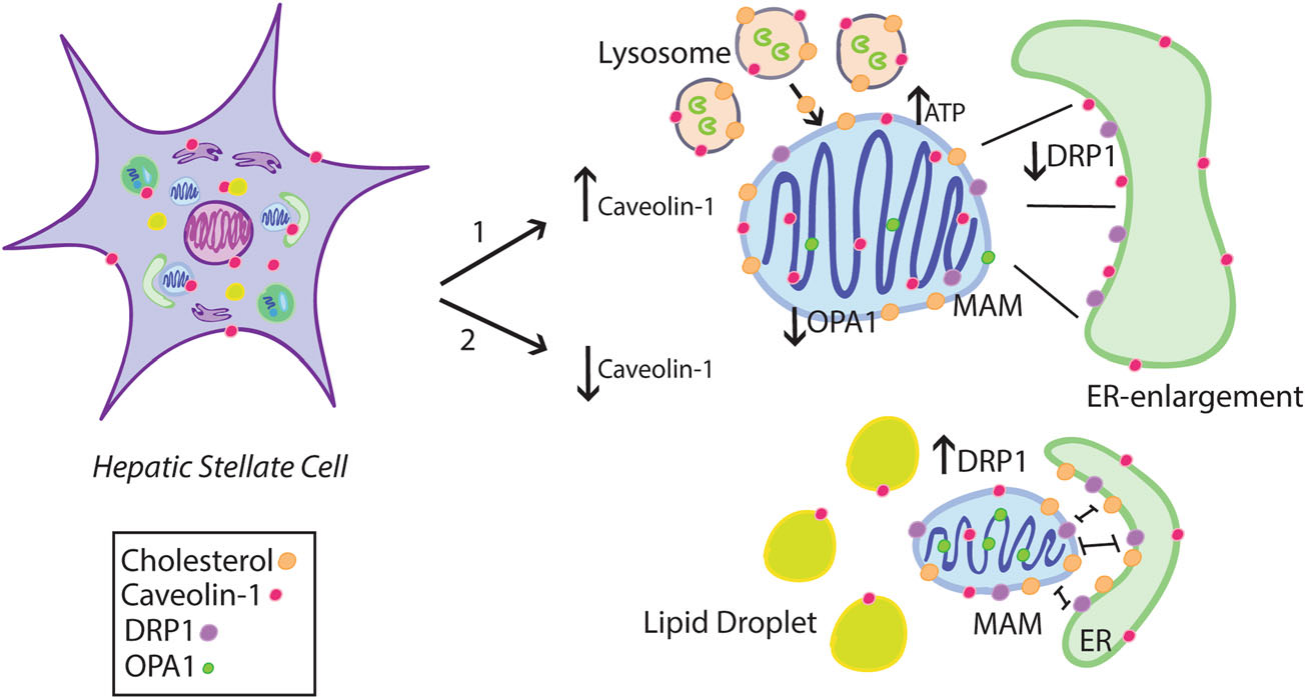
A schematic view of mitochondrial plasticity of HSC in exogenous expression (1) or knockdown (2) of Caveolin‐1. Caveolin‐1 expression changes mitochondrial size, dynamics, cholesterol content, ER‐mitochondria interactions, and lysosomal activity in HSCs. DRP1, dynamin 1‐like protein; ER, endoplasmic reticulum; HSC, hepatic stellate cell; MAM, mitochondrial associated membrane; OPA1, optic atrophy protein 1.

We found that exogenous expression of Cav‐1 triggered an increase in lysosomal bioactivity, ER enlargement, and changes in inter‐organelle communication, a mechanism that could drive HSC to activation (Luo et al., 2018; Tsuchida & Friedman, 2017). Lysosomes are a source of lipoprotein‐derived cholesterol and form contact with mitochondria to deliver the cholesterol to mitochondria (Silva et al., 2020). Cav‐1 regulates the levels of mitochondrial cholesterol by promoting cholesterol efflux from the ER through MAM structural platform, affecting mitochondrial function, morphology, and bioenergetic response (Fridolfsson et al., 2014; Rieusset, 2018). In these processes, Cav‐1 moves to and from the cytoplasm surface of lysosomes during intracellular cholesterol trafficking (Fridolfsson et al., 2014). Additionally, we saw a correlation between the mitochondrial cholesterol, mitochondrial area, and perimeter. These results bring to us a notion that the increase of mitochondrial cholesterol content accompanies the increase of mitochondrial area and perimeter, suggesting that there is no mitochondrial dysfunction by exogenous expression of Cav‐1. Another interesting result from the correlations with mitochondrial cholesterol was with Cav‐1, DRP1 expression, ER‐OMM distance, and LYSR. These results led us to think that exogenous expression and knockdown of Cav‐1 can modulate the mitochondrial dynamics and organization. One possible mechanism is that exogenous expression of Cav‐1 could provide an alternative route for mitochondrial cholesterol that enters through the connection with lysosomes instead of ER‐mitochondria approximation (Höglinger et al., 2019). However, whether Cav‐1 influences the transport of mitochondrial cholesterol through the lysosome contacts in HSCs is still unknown. On the other hand, knockdown of Cav‐1 seems to distribute the mitochondrial cholesterol majority through MAM proximity. Also, the high presence of LDs can suggest a profile less activated by HSCs. From a physiological perspective, considering also our previously published results (Ilha et al., 2019), the oxidative metabolism alterations induced by exogenous expression of Cav‐1 could be associated with cell biological changes and an increase in mitochondrial plasticity during the HSC activation. Based on our results, it is possible to suggest that Cav‐1 has a relevant function in the pathophysiology of chronic liver diseases through an important physiological role for mitochondria dynamics, lysosomes, ER shape, and MAM during the activation process of HSC (Figure 5).

In conclusion, we observed that Cav‐1 can modulate mitochondrial plasticity, flexibility, and function during HSCs activation. Future studies are required to understand the mechanistic role and how Cav‐1 can influence mitochondrial plasticity during the process of HSC transdifferentiation. Altogether, our findings suggest that Cav‐1 is a promising candidate for further studies aimed at identifying new treatments for chronic liver diseases.

## AUTHOR CONTRIBUTIONS

**Mariana Ilha:** Conceptualization, methodology, validation, data curation, formal analysis, investigation, visualization, and writing – original draft. **Leo A. Meira Martins**: Methodology, investigation, formal analysis, and writing – review and editing. **Ketlen da Silveira Moraes**: Methodology and validation. **Camila K. Dias**: Methodology, validation, investigation, and writing – review and editing. **Marcos P. Thomé**: Methodology, validation, and investigation. **Fernanda Petry**: Methodology, validation, and investigation. **Francieli Rohden**: Methodology, validation, and investigation. **Radovan Borojevic**: Resources. **Vera M. T. Trindade**: Investigation, resources, writing – review and editing, and supervision. **Fábio Klamt**: Investigation, resources, funding acquisition, writing – review and editing, and supervision. **Florência Barbé‐Tuana**: Investigation, resources, writing – review and editing, and supervision. **Guido Lenz**: Investigation, resources, writing – review and editing, and supervision. **Fátima C. R. Guma:** Conceptualization, investigation, resources, writing – review and editing, supervision, funding acquisition, and project administration. All the authors studied and approved the final manuscript.

## CONFLICT OF INTEREST

The authors declare no conflict of interest.

## Supporting information

Supporting information.

## Data Availability

All data is available within the manuscript and in the supplementary information.
