# Supplementary material for: Caveolin‐1 influences mitochondrial plasticity and function in hepatic stellate cell activation
Source: Cell Biol Int. 2022 Aug 16;46(11):1787–800. doi: 10.1002/cbin.11876 (PMC9804617; doi:10.1002/cbin.11876)
Supplement: Supplementary file 1 — Supporting information. [file CBIN-46-1787-s001.docx]

**Supplementary figures**


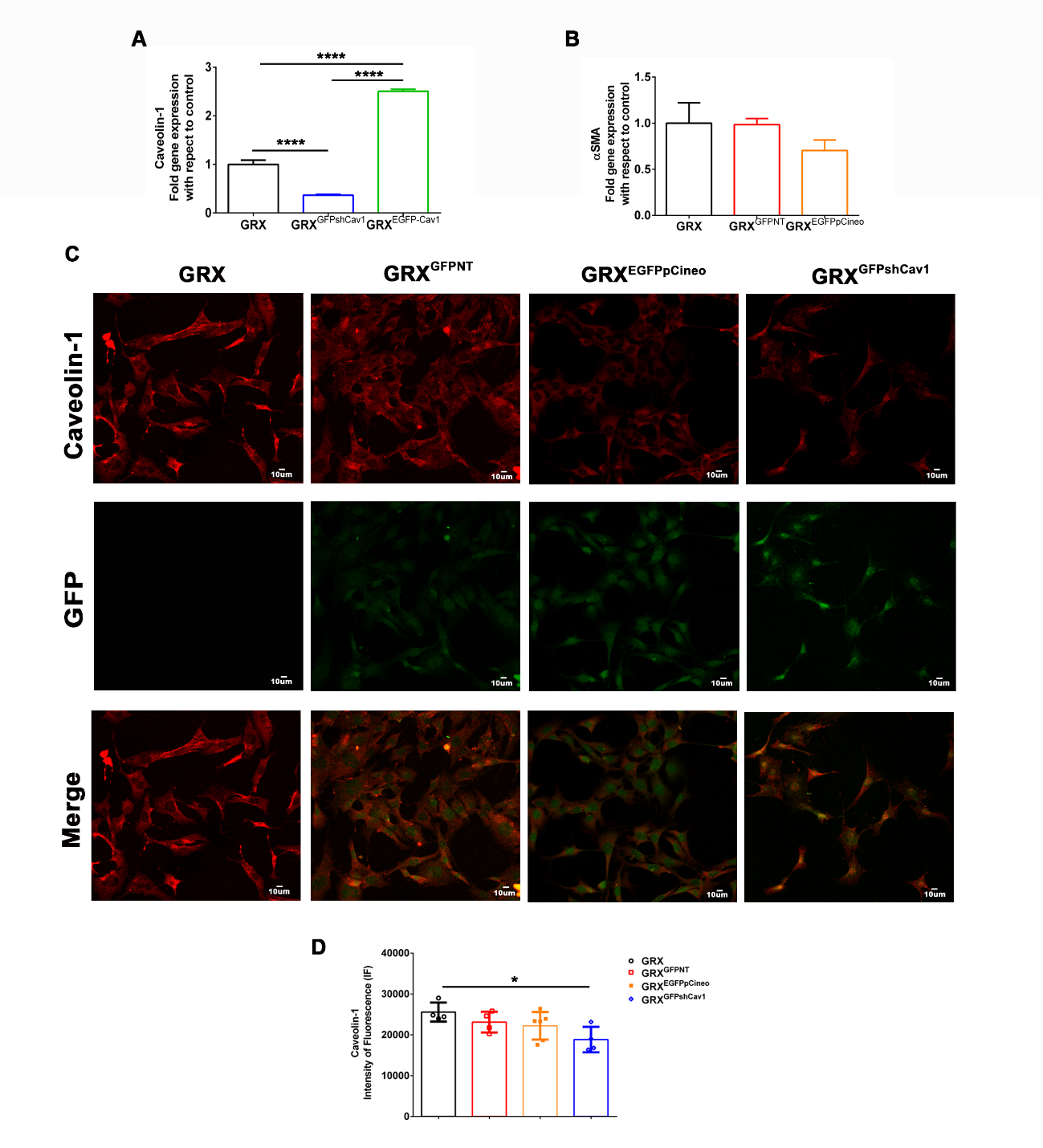


**Supplementary Fig. S1:** Stable shRNA silencing of Caveolin-1 in GRX cell line. **A)** GRX^EGFP-Cav1^ showed an increase of 150% in Cav-1 mRNA expression. The knockdown of Cav-1 led to a significant reduction of 64% in Cav-1 mRNA expression. **B)** The presence of GFP or EGFP did not differ αSMA mRNA expression in the GRX^GFPNT^ and GRX^EGFPpCineo^ controls. **C)** Cells were immunolabelled for Caveolin-1 using monoclonal anti-Cav-1 primary antibody followed by Alexa Fluor 555 secondary antibody. GFP confirms the success of the lentiviral knockdown of Cav-1. Scale bar 10µm. **D)** GRX^GFPshCav1^ showed a decrease in the intensity of fluorescence (IF) of Cav-1 protein. Graphs values are means ± SD (n=3 experiments, **P*< 0.05 and *****P*< 0.0001 such as indicated by one-way ANOVA followed by Bonferroni´s post-test)


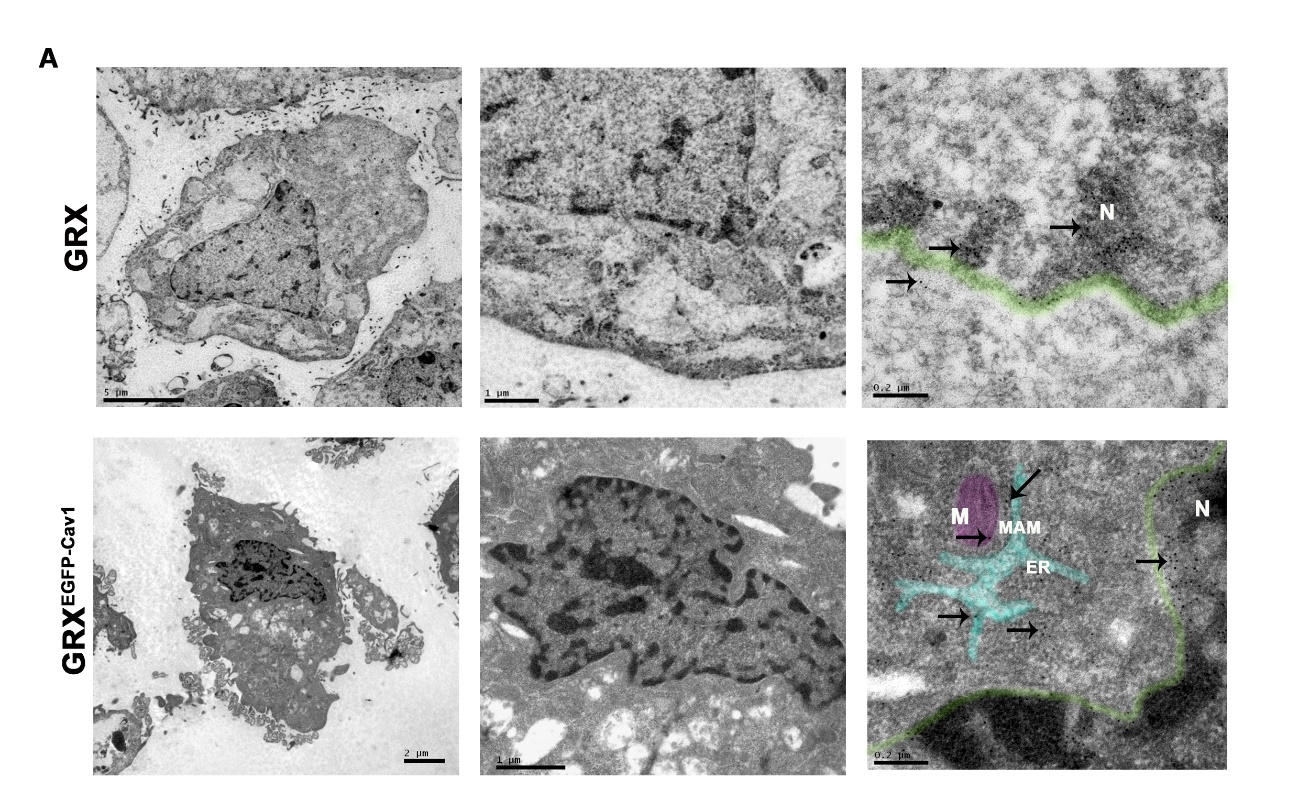


**Supplementary Fig. S2:** Transmission electron images showing Cav-1 labeling in the hepatic stellate cell, GRX, and GRX^EGFP-Cav1^ cells. **A)** TEM Representative images of cells stained with immunogold particles of 9 nm indicating the Cav-1 labeling in GRX and GRX^EGFP-Cav1^. Black arrows in GRX showed that Cav-1 particles are mostly present in the nucleus (N) and cytoplasm. For GRX^EGFP-Cav1^, the black arrows showed Cav-1 gold particles in the inner and outer mitochondria membrane (M, pink), mitochondria-associated membranes (MAM), ER (ER, cyan blue), and nuclear membrane (N, green). Magnification 5, 2, 1 and 0,2 µm. n=4 experiments, at least 15 images were acquired by the group
